# Alien and native tree species having extrafloral nectaries as favorite hunting area for arboreal endemic Philippine tiger beetles (Coleoptera: Cicindelidae) in human‐disturbed habitat in Lanao del Sur Province, Mindanao, Philippines

**DOI:** 10.1002/ece3.7149

**Published:** 2021-01-05

**Authors:** Jalanie S. Marohomsalic, Olga Macas Nuñeza, Marek Michalski, Jürgen Wiesner, Radomir Jaskuła

**Affiliations:** ^1^ Department of Biological Sciences College of Science and Mathematics Mindanao State University‐Iligan Institute of Technology Iligan City Philippines; ^2^ Department of Experimental Zoology and Evolutionary Biology Faculty of Biology and Environmental Protection University of Lodz Lodz Poland; ^3^ Wolfsburg Germany; ^4^ Department of Invertebrate Zoology and Hydrobiology Faculty of Biology and Environmental Protection University of Lodz Lodz Poland

**Keywords:** insect interactions, invasive trees, Mindanao, *Neocollyris*, Philippines, plant, *Tricondyla*

## Abstract

To document a relation between abundance of arboreal, predatory tiger beetles, their ant prey, and extrafloral nectaries attracting the ants, we gathered data from more than 10 species of native and introduced trees and large, tree‐like perennial plants in Lanao del Sur Province, Mindanao, Philippines. All specimens of tiger beetles (two *Tricondyla* and two *Neocollyris* species, all endemic to the country) were noted on five tree species characterized by presence of extrafloral nectaries, including three alien/invasive and two native ones. Invasive *Spathodea campanulata* and native *Hibiscus tiliaceus* were the most inhabited ones (respectively, 56% and 19% of beetles). Presence of tiger beetles on these trees most probably depends on high abundance of ants, which are typical prey for arboreal Cicindelidae, while occurrence of ants can result from presence of extrafloral nectaries on different parts of the plants. This suggests a new mutualistic insect–plant interaction between native and invasive species.

## Graphical abstract

Arboreal tiger beetle species used invasive and native trees having extrafloral nectaries as preferred place for hunting on ants, the insects which are attracted to these trees because of presence of sweet substances produced in these elements. Based on these observations, we suggest a new mutualistic insect–plant interaction between native and invasive species. In our opinion, this is the first case of such interactions noted between tiger beetles and invasive trees both in the Philippines and in the entire area of the southeastern Asia.

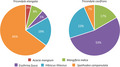

## 1 INTRODUCTION

In the Philippines, more than 40 alien plant taxa have been noted including at least 11 tree species (Global Invasive Species Database, 2019). They originate from different regions of the world and were introduced for their utilitarian or ornamental values. As occurrence and distribution of some of such tree species can be supported by different animals, for example, flower pollinators or seed collectors (Corlett, 1998; Forget & Milleron, 1991; Nathan et al., 2008; Wenny & Levey, 1998), some of them started to occur in many seminatural and natural areas. Although there is a lack of detailed studies focused on majority of alien tree species in the Philippines, usually influence of such alien/invasive plants on natural environment seems to be negative (e.g., Dostál et al., 2013; Harvey & Fortuna, 2012). On the other hand, every single tree (including invasive species) constitutes unique microecosystem occupied by numerous animals, what is especially well seen in case of insect diversity in the tropics (Erwin, 1982; Erwin & Scott, 1981).

Tiger beetles (Coleoptera: Cicindelidae) are group of predatory insects hunting mainly on various small invertebrates (Pearson & Vogler, 2001; Rewicz & Jaskuła, 2018). In the Philippines, more than 160 taxa of tiger beetles have been noted (Anichtchenko & Medina, 2019, 2020; Cabras et al., 2016; Dheurle, 2016, 2019; Medina et al., 2019, 2020; Wiesner & Dheurle, 2018; Zettel & Wiesner, 2018) including 14 *Tricondyla* Latreille et Dejean, 1822 and 29 *Neocollyris* Horn, 1901 species of which almost 90% are endemic to this country (Cabras et al., 2016). Although there is no detailed study devoted to prey preferences of arboreal *Tricondyla* or *Neocollyris* species, unspecified ant species are suggested as one of the most important types of prey both for larvae and adults of these ant‐mimicking tiger beetles (Naviaux, 2002; Pearson & Vogler, 2001; Trautner & Schawaller, 1996).

It is estimated that extrafloral nectaries occur in 1%–2% of terrestrial plant species. These highly diversified structures, located at almost every above‐ground organ of plants, produce aqueous solution of carbohydrates and other compounds, similar to “classic” nectar produced in flowers (Weber & Keeler, 2013). As different species of ants (Formicidae) are the most common insects utilizing this source of food, it is generally accepted that occurrence of extrafloral nectaries is evidence of coevolution between plant and ants. There are three main models of this coevolution: protection, flower distraction, and ant distraction. Probably, first of them is the most common; in this model, the ants protect plant against herbivores, obtaining in return “reward” from the plant. Two other models assume that plant with help of extrafloral nectaries distracts ants from flowers, where they may affect pollination, or from their herbivorous trophobionts (Del‐Claro et al., 2016).

## 2 STUDY AREA, MATERIAL, AND METHODS

**FIGURE 1:**
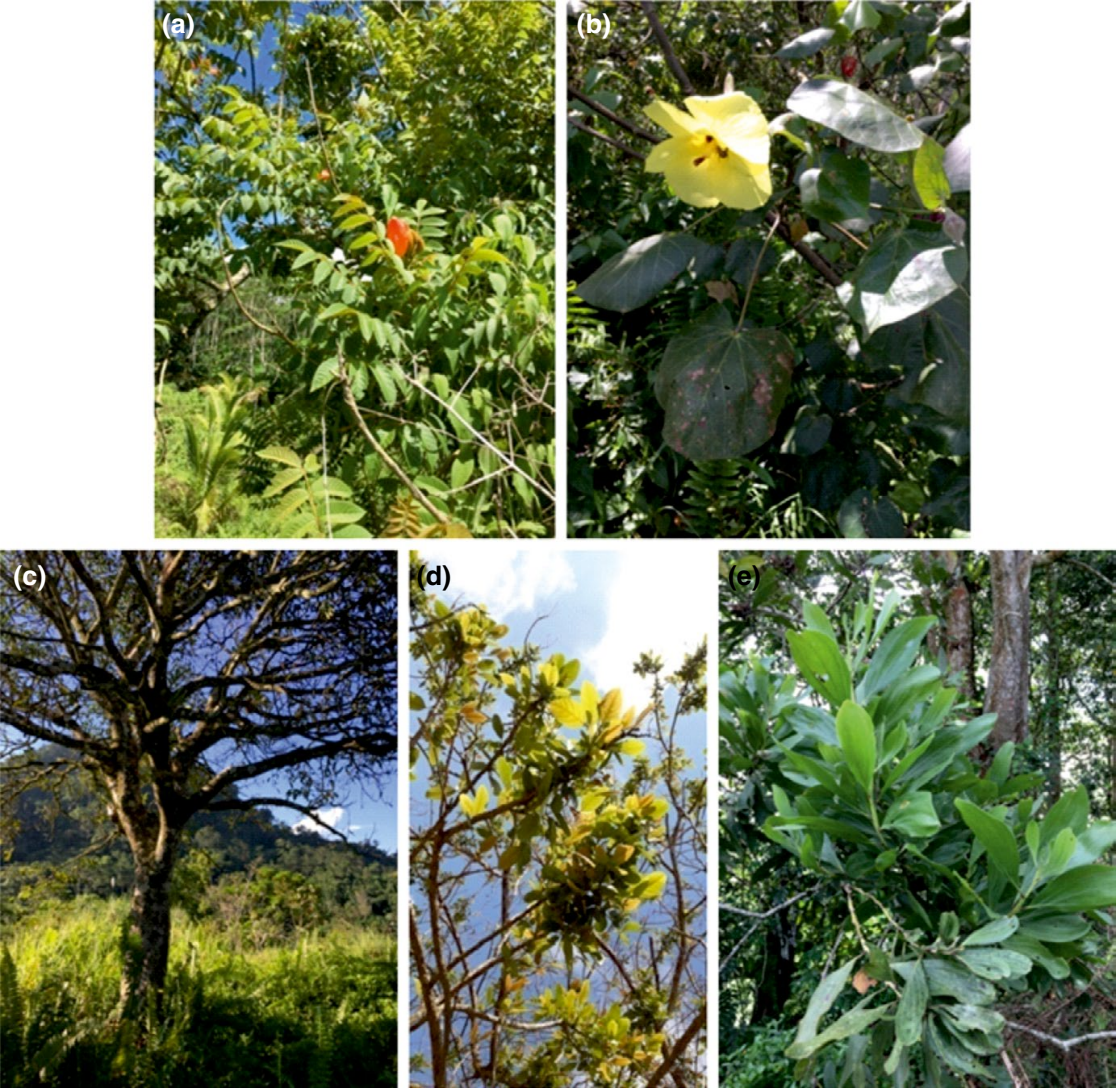
Tree species inhabited by arboreal tiger beetles in Lanao del Sur Province, Mindanao Philippines: (a) *Spathodea campanulata, (b) *Hibiscus tiliaceus*, (c) *Erythrina fusca*, (d) *Mangifera indica, (e) *Acacia mangium (phot. J. S. Marohomsalic). Alien/invasive species were marked with asterisk (*)

Adult tiger beetles were collected through opportunistic sampling from tree trunks, branches, and leaves using entomological hand net from 9 April to 4 August 2019 in the municipality of Masiu, Saguiran, and Marawi City, Lanao del Sur Province, Mindanao, Philippines. A total of 10 sampling sites were established in these areas (Table 1). Some sampling sites were visited several times, while others surveyed once due to time constraints and availability. During every visit in every sampling site, all specimens of every tree species present in particular areas were checked (Table 1). Sampling sites were located in human‐disturbed habitat close to human settlements or even in the villages. In total, over 10 tree and large, tree‐like perennial species have been checked, including three taxa alien and/or invasive for the Philippines (Figure 1): *Spathodea campanulata* P. Beauv. (native to Africa, one of the most invasive tree species in the world), *Mangifera indica* L. (native to continental south‐east Asia), and *Acacia mangium* Willd. (native to Australia, Papua, and Moluccas). Moreover seven native taxa, *Erythrina fusca* Lour., *Hibiscus tiliaceus* L., *Artocarpus heterophyllus* Lam., *A. odoratissimus* Blanco, *Cocos nucifera* L., *Musa* spp., and *Bambusa* spp., have been checked as well. According to literature (Elias & Prance, 1978; Feinsinger & Swarm, 1978; Peng, 2015; Savage et al., 2009; Seibert, 1948; Sugiura et al., 2006; Zhang et al., 2012; Zimmermann, 1932), all invasive species and first two of the mentioned native taxa can be characterized by presence of extrafloral nectaries.

**TABLE 1:** Sampling sites in Lanao del Sur Province and number of trees and large, tree‐like perennial plants checked according to arboreal tiger beetle species

	Locality	Sampling period	Trees species						
			*#Spathodea campanulata	*#Hibiscus tiliaceus*	*#Erythrina fusca*	*#Mangifera indica	*#Acacia mangium	All other species	Total
S1	Saguiran: Barangay Pawak, 08°03.399′N 124°16.024′E, 534 m a.s.l., open, riverine, mountainous area with forest patches of trees	09.04.2019. 25.05.2019. 06.06.2019.	2	0	0	0	0	3	5
S2	Saguiran: Barangay Mipaga, 08°02.314′*N* 124°16.922′E, 655 m a.s.l., open, riverine, mountainous area with forest patches of trees	17.04.2019. 26.05.2019. 03.08.2019.	5	1	2	0	0	4	12
S3	Saguiran: Barangay Sunggod, 08°03.408′N 124°16.317′E, 544 m a.s.l, open, riverine, mountainous area with forest patches of trees	19.04.2019. 26.05.2019.	0	0	1	0	0	4	5
S4	Marawi City: Barangay Mipaga, 08°01.740′N 124°17.072′E, 661 m a.s.l., open, riverine, mountainous area with forest patches of trees mixed with agricultural field	27.05.2019. 04.06.2019. 27.05.2019.	5	0	0	2	0	3	10
S5	Marawi City: Barangay Rorogagus Proper, 08°01.445′N 124°17.131′E, 671 m a.sl., open, riverine, mountainous area with forest patches of trees mixed with agricultural field	03.06.2019. 04.06.2019. 16.06.2019.	0	1	0	2	3	2	8
S6	Marawi City: Barangay Guimba, 08°01.550′N 124°17.596′E, 716 m a.s.l, open, mountainous area with forest patches of trees	13.06.2019. 16.06.2019.	3	9	0	0	1	6	19
S7	Marawi City: Barangay, Lilod Saduc, 08°00.762′N 124°17.895′E, 734 m a.s.l, open, riverine, mountainous area with forest patches of trees	17.06.2019. 18.07.2019.	1	0	1	1	1	5	9
S8	Marawi City: Barangay Caloocan, 07°59.279′N 124°18.369′E, 714 m a.s.l, open area with patches of trees on the bank of lake	19.06.2019.	1	0	3	0	0	3	7
	Total	17	11	7	5	5	30	75	

Note:

All other species means: *Artocarpus heterophyllus*, *A. odoratissimus*, *Cocos nucifera*, *Musa* spp., and *Bambusa* spp. Alien/invasive taxa are indicated by asterisk (*), while species having extrafloral nectaries are marked by hash (#).

Insects were collected usually between 7 a.m. and noon and from 1 p.m. to 5 p.m. as it is the time of highest activity of adult tiger beetles (Pearson & Vogler, 2001). Material was preserved in 96% ethanol and is currently deposited in collections of the first and last author.

Numbers of tiger beetles noted on alien/invasive and native tree species were tested for statistical significant differences applying Mann–Whitney U tests, while in case of tree preferences of *Tricondyla* species, chi‐square tests were conducted using TIBCO Statistica v. 13.3 software.

## 3 RESULTS

Tiger beetles were found only on plants belonging to five species (45 individuals or 60% of checked plants), including all alien and/or invasive (Figure 1, Table 1): *Spathodea campanulata* P. Beauv.*, Mangifera indica* L., and *Acacia mangium* Willd., and two native tree species: *Erythrina fusca* Lour and *Hibiscus tiliaceus* L. From all other tree species or tree‐like perennial plants, no tiger beetles have been recorded. In total, 228 tiger beetles belonging to four species known as Philippine endemics (Cabras et al., 2016) were noted: *Tricondyla* (*Stenotricondyla*) *cavifrons* Schaum, 1862—30 specimens, *T*. (*Tricondyla*) *elongata* W. Horn, 1906—193 individuals, *Neocollyris* (*Heterocollyris*) *similior* (Horn, 1893)—1 beetle, and *N*. (*Neocollyris*) cf. *albitarsis* (Erichson, 1834)—4 specimens. *Tricondyla* beetles were observed on tree branches, while *Neocollyris* were recorded only on the leaves, all of them were searching and/or hunting on ants and/or other small arthropods. All mentioned tiger beetle species were noted from Lanao del Sur Province for the first time.

In total, 78% recorded tiger beetles were collected from alien/invasive tree species while only 22% from native ones. All beetles were noted on tree species characterized by presence of extrafloral nectaries on their different parts (91% if *Mangifera indica* is excluded as in case of this species presence of such nectaries is sometimes questioned). Invasive *Spathodea campanulata* was the most preferred tree with a total of 56% of Cicindelidae (128 individuals) noted in the study, including, respectively, 125 specimens of *Tricondyla elongata* (65% individuals of the species), two individuals of *T*. *cavifrons* (7% of the species), and a single *Neocollyris* cf. *albitarsis* (25% of the species). Native *Hibiscus tiliaceus* and *Erythrina fusca* were recorded as the second and the third preferred tree by tiger beetles (respectively, 19% and 13% of all collected specimens) followed by alien taxa *Mangifera indica* L. (9%), and *Acacia mangium* (4%). The result of Mann–Whitney *U* tests shown nonsignificant differences between number of tiger beetles recorded on alien/invasive and native trees.

**FIGURE 2:**
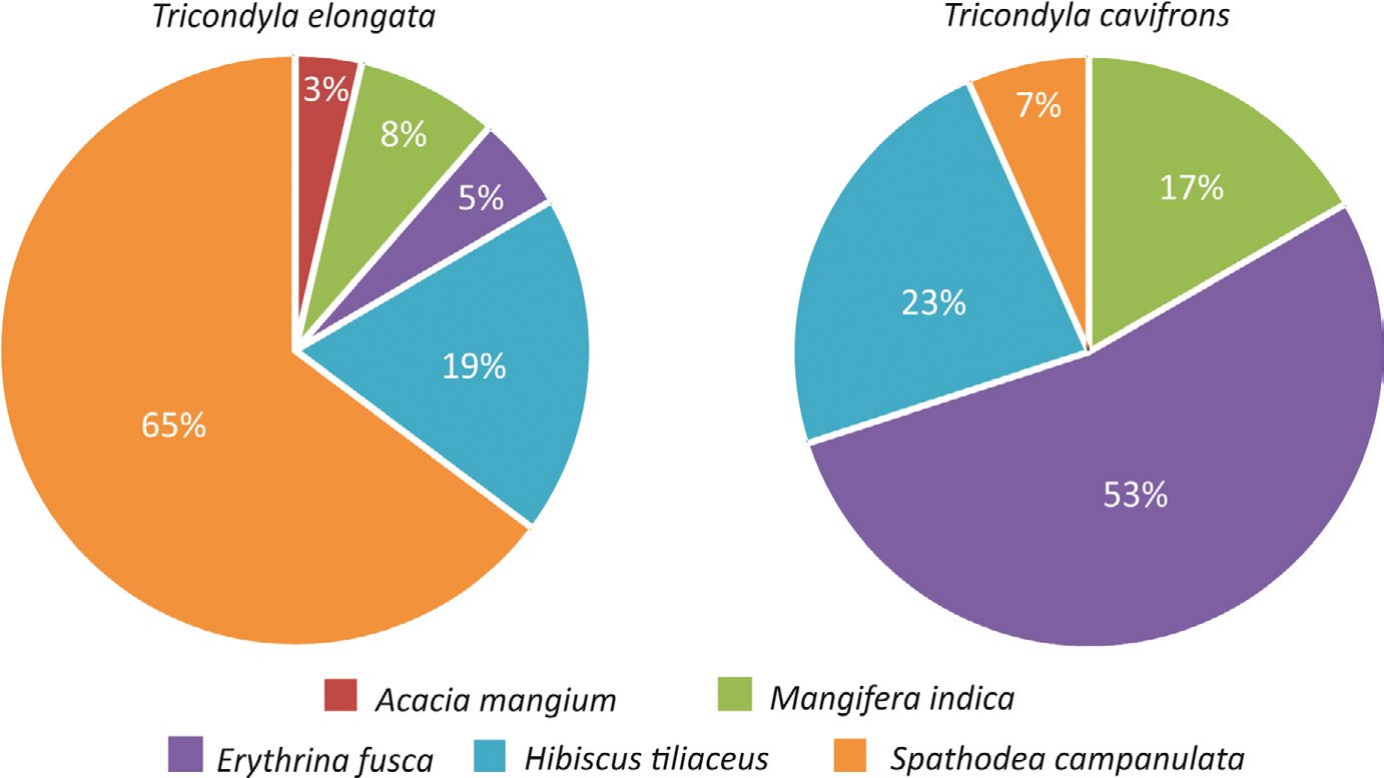
Tree preferences in *Tricondyla* tiger beetle species in studied habitats in Lanao sel Sur Province, Mindanao, Philippines

Figure 2 shows that *Tricondyla elongata* was observed at five tree species; however, *Spathodea campanulata* and *Hibiscus tiliaceus* seemed to be the favorable for this species (respectively, 65% and 19% of noted specimens). Three other species (*Erythrina fusca*, *Mangifera indica*, and *Acacia mangium*) were visited by less than 10% tiger beetles each. In contrast, *Tricondyla cavifrons* was found on four tree species, especially on tree trunks and branches of *Erythrina fusca* (53%), *Hibiscus tiliaceus* (23%), and *Mangifera indica* (17%). Only 7% of hunting specimens of this species was noted on *Spathodea campanulata*. The result of chi‐square test shown significant differences between these two *Tricondyla* species according to visited tree species (*χ* ^2^ = 33.92, *p* < .001). In case of both *Neocollyris* species, it is rather difficult to estimate tree preferences as only single individuals were noted during this study.

## 4 DISCUSSION

Generally it is known that cicindelids are insects with narrow or even very narrow habitat specialization (e.g., Freitag, 1979; Ganeshaiah & Belavadi, 1986; Jaskuła, 2011, 2015; Jaskuła & Płóciennik, 2020; Jaskuła et al., 2019; Knisley & Pearson, 1984; Pearson, 1984; Satoh & Hori, 2005; Satoh et al., 2006; Schultz & Hadley, 1987; Zerm & Adis, 2001). This specialization makes the group very sensitive for environmental changes especially resulting from human activity (Arndt et al., 2005; Knisley, 2010; Knisley and Hill, 1992). Although as for now there is a lack of such data for Philippine arboreal tiger beetles, similar results are obtained in case of epigeic species studied in this country (Jaskuła et al., in prep.). Moreover, there are numerous published examples of negative impact of alien/invasive plant species on animal/insect diversity in areas where they were introduced (e.g., Dostál et al., 2013; Harvey & Fortuna, 2012), even if gradually some of insect species start to use alien plants as food or habitat (e.g., Brändle et al., 2008; Karolewski et al., 2014; Meijer et al., 2012; Novotny et al., 2003). Taking all these facts into consideration, one can note that high abundance of endemic predatory tiger beetles (Cabras et al., 2016) occurring not only on native but also on invasive/alien tree species in Lanao del Sur is unusual and surprising, until we will pay attention on biology and ecology of both arboreal Cicindelidae and tree species on which these beetles were recorded in current study. Both *Tricondyla* and *Neocollyris* tiger beetles are known as day active predators hunting on trees and bushes on different small insects, with preference of ants (Clausen, 1940; Naviaux, 2002; Trautner & Schawaller, 1996; R. Jaskuła—personal observation). On the other hand, most (all?) tree species which were recorded by us as attractive for arboreal tiger beetles can be characterized by presence of extrafloral nectaries on different parts of the plants (Weber et al., 2020). For example, *Spathodea campanulata* has nectaries on fruits, sides of midrib, surface of the leaves, flower pedicels, and external surface of the petals (Elias & Prance, 1978; Seibert, 1948; Zimmermann, 1932), and in *Hibiscus tiliaceus*, they are present on leaf base, main nerve, and sepals (Savage et al., 2009; Sugiura et al., 2006; Zimmermann, 1932), while *Acacia mangium* has them on basal parts of leaf stalks (Zhang et al., 2012). In *Erythrina fusca*, their presence was unspecified (Feinsinger & Swarm, 1978; Savage et al., 2009) but other species classified in this genus have external nectaries on stipules and on the sepals (Zimmermann, 1932). In case of *Mangifera indica*, some sources state that nectaries are present on young leaves, petioles, and developing fruits (Peng, 2015) while other suggest that they are absent in the species (Weber et al., 2020).

Presence of extrafloral nectaries on mentioned above plants was not studied by us in details, but high number of insects, especially different ant species, was noted on all these trees checked in all study sites. Although we are not able to confirm whether these insects were using extrafloral nectaries of *Spathodea campanulata*, *Hibiscus tiliaceus*, *Acacia mangium*, *Erythrina fusca*, and/or *Mangifera indica* as food source, numerous examples from different regions of the world clearly show that high diversity and abundance of Formicidae on plant species having such structures is a typical phenomenon (e.g., Aguirre‐Jaimes et al., 2018; Fonseca‐Romero et al., 2019; Fotso et al., 2015; Giuliani et al., 2019; Sanz‐Veiga et al., 2017). As it was noted in the literature, ants collect food from extrafloral nectaries and at the same time they can protect the plant against herbivorous insects (Lin et al., 2018), so both partners of such mutualistic relationship have benefits. On the other hand, it can be expected that high number of ants can result in higher number of predators specializing in such type of prey, like arboreal tiger beetles, especially *Tricondyla* species. Although no details about Formicidae species were provided by the authors, we believe that most probably such example comes also from the paper by Abeywardhana et al. (2019) who noted *T. gounelli* Horn, 1900 on *Anacardium occidentale*. This tree species has extrafloral nectaries on leaves, flowers, inflorescences, and fruits, what makes this plant very attractive for ants and, consequently, for ant predators including arboreal tiger beetles. Although definitely future studies are necessary to confirm this hypothesis, the described situation clearly suggests that at least some arboreal Cicindelidae can benefit from presence of alien/invasive tree species in human‐changed habitats because of high concentration of their potential prey. As *Hibiscus tiliaceus* and *Erythrina fusca*, which are native to the Philippines, were the second and the third tree species according to number of tiger beetles noted in this study, we suppose that presence of these insects on trees having extrafloral nectaries is not fully a novel hunting adaptation (even if never studied before), but can suggest that at least some arboreal tiger beetles can easily adapt to environmental changes like is was observed earlier in other representatives of this family (e.g., Cabrera et al., 2019; Gilbert, 1997; Rewicz & Jaskuła, 2018; Riggins & Hoback, 2005). Moreover, our results clearly suggest that presence of alien/invasive tree species, even if generally it has negative impact on biodiversity (e.g., Dostál et al., 2013; Harvey & Fortuna, 2012), occasionally can support local populations of some native or even endemic taxa, especially in highly human‐disturbed habitats.

## CONFLICT OF INTEREST

All authors declare no conflict of interest including any financial, personal, or other relationships with other people or organizations within 3 years of beginning the submitted work that could inappropriately influence, or be perceived to influence, their work.

## AUTHOR CONTRIBUTION

**Jalanie S. Marohomsalic:** Conceptualization (equal); Data curation (lead); Formal analysis (lead); Funding acquisition (equal); Investigation (lead); Methodology (equal); Resources (equal); Validation (equal); Visualization (equal); Writing‐original draft (equal); Writing‐review & editing (equal). **Olga Macas Nuñeza:** Conceptualization (equal); Data curation (equal); Formal analysis (equal); Funding acquisition (lead); Investigation (supporting); Methodology (supporting); Supervision (equal); Validation (equal); Writing‐original draft (supporting); Writing‐review & editing (supporting). **Marek Michalski:** Conceptualization (equal); Formal analysis (equal); Writing‐original draft (equal); Writing‐review & editing (equal). **Jürgen Wiesner:** Formal analysis (equal); Writing‐review & editing (equal). **Radomir Jaskuła:** Conceptualization (lead); Formal analysis (equal); Investigation (supporting); Methodology (equal); Project administration (lead); Resources (equal); Software (equal); Supervision (lead); Validation (equal); Visualization (equal); Writing‐original draft (lead); Writing‐review & editing (lead).

## Data Availability

The data are available in the Dryad database under the following link: https://doi.org/10.5061/dryad.nk98sf7r4
